# Radiation safety measures in diagnostic nuclear medicine, based on the potential radiation dose emitted by radioactive patients

**DOI:** 10.1590/0100-3984.2022.0064

**Published:** 2023

**Authors:** José Willegaignon, Samantha Cristina Pereira Fernandes, Rogério Alexandre Pelissoni, George Barbério Coura-Filho, Marcelo Tatit Sapienza, Carlos Alberto Buchpiguel

**Affiliations:** 1 Instituto do Câncer do Estado de São Paulo (Icesp), Hospital das Clínicas da Faculdade de Medicina da Universidade de São Paulo (HC-FMUSP), São Paulo, SP, Brazil; 2 Departamento de Radiologia, Faculdade de Medicina da Universidade de São Paulo (FMUSP), São Paulo, SP, Brazil

**Keywords:** Nuclear medicine, Diagnostic imaging, Radiation exposure, Radiotherapy dosage, Radiation protection, Medicina nuclear, Diagnóstico por imagem, Exposição à, radiação, Dosagem radioterapêutica, Proteção radiológica

## Abstract

**Objective:**

To measure the potential radiation dose emitted by patients who have recently undergone diagnostic nuclear medicine procedures, in order to establish optimal radiation safety measures for such procedures.

**Materials and Methods:**

We evaluated the radiation doses emitted by 175 adult patients in whom technetium-99m, iodine-131, and fluorine-18 radionuclides were administered for bone, kidney, heart, brain, and whole-body scans, as measured with a radiation detector. Those values served as the basis for evaluating whole-body radiopharmaceutical clearance, as well as the risk for the exposure of others to radiation, depending on the time elapsed since administration of the radiopharmaceutical.

**Results:**

The mean time to clearance of the radiopharmaceuticals administered, expressed as the effective half-life, ranged from 1.18 ± 0.30 h to 11.41 ± 0.02 h, and the mean maximum cumulative radiation dose at 1.0 m from the patients was 149.74 ± 56.72 µSv. Even at a distance of 0.5 m, the cumulative dose was found to be only half and one tenth of the limits established for exposure of the general public and family members/caregivers (1.0 mSv and 5.0 mSv per episode, respectively).

**Conclusion:**

Cumulative radiation doses emitted by patients immediately after diagnostic nuclear medicine procedures are considerably lower than the limits established by the International Commission on Radiological Protection and the International Atomic Energy Agency, and precautionary measures to avoid radiation exposure are therefore not required after such procedures.

## Abstract (pt)

**Objetivo:**

O objetivo deste trabalho foi levantar o potencial de dose de radiação emitida por pacientes em procedimentos diagnósticos, visando a estabelecer cuidados de radioproteção mais otimizados.

**Materiais e Métodos:**

Taxas de dose de radiação emitidas por 175 pacientes administrados com os radionuclídeos ^99m^Tc, ^131^I e ^18^F para cintilografias óssea, renal, cardíaca, cerebral e corpo inteiro, foram mensuradas com um detector de radiação, servindo para avaliar o clareamento do radiofármaco no organismo e risco de exposição após administração dos radiofármacos.

**Resultados:**

O clareamento, representado pela meia-vida efetiva, variou de 1,18 ± 0,30 h até 11,41 ± 0,02 h e a dose de radiação máxima acumulada oferecida pelos pacientes a 1,0 m foi de 149,74 ± 56,72 µSv. Mesmo para distâncias de 0,5 m, as doses estimadas foram, respectivamente, duas e dez vezes inferiores ao nível de restrição para o público geral (1,0 mSv) e exposição médica (5,0 mSv/episódio).

**Conclusão:**

Doses de radiação oferecidas por pacientes em procedimentos diagnósticos são inferiores aos níveis de restrição recomendados pela International Commission on Radiological Protection e International Atomic Energy Agency, e assim, cuidados de radioproteção são geralmente desnecessários.

## INTRODUCTION

After receiving a radiopharmaceutical during diagnostic or therapeutic procedures, patients become (temporarily) radioactive and can expose others to ionizing radiation for some time. The radionuclides typically administered during therapeutic nuclear medicine procedures have high levels of activity, and radiation protection measures are therefore taken to monitor and reduce radiation exposure, not only of the patients themselves but also of health care professionals and others, such as family members, caregivers, and work colleagues^(1,2)^. However, those measures may be foregone during diagnostic procedures, which require the administration of much smaller quantities of radionuclides, because whole-body excretion of the radionuclides is more rapid and the level of whole-body radiation emitted is lower^(3-7)^. Unfortunately, there have been few studies investigating the real potential for exposure from radioactive patients after such diagnostic procedures.

Apart from other associated risks, the widespread application of nuclear medicine techniques for diagnosing human diseases could lead to an increase in the incidence of radiation exposure of medical staff and the public. Based on the factors mentioned above, the aim of this study was to determine the potential risks of radioactive patients exposing health professionals and others after having received radiopharmaceuticals during diagnostic nuclear medicine procedures. Our findings could lay the groundwork for the establishment of optimized radiation safety measures to be taken under those circumstances.

## MATERIALS AND METHODS

### Nuclear medicine diagnostic procedures and patients

The nuclear medicine diagnostic procedures evaluated in this study were selected from among the various examinations available at our facility. Nine were selected for patient eligibility: bone scintigraphy with technetium-99m-methylene diphosphonate (^99m^Tc-MDP); positron emission tomography (PET) for bone scanning with fluorine-18-sodium fluoride (^18^F-NaF); static renal scintigraphy with ^99m^Tc-dimercaptosuccinic acid (^99m^Tc-DMSA); dynamic renal scintigraphy with ^99m^Tc-diethylenetriamine pentaacetic acid (^99m^Tc-DTPA); brain scintigraphy with ^99m^Tc-methoxyisobutylisonitrile (^99m^Tc-MIBI); myocardial perfusion study with ^99m^Tc-MIBI; whole-body scintigraphy with ^99m^Tc-MIBI and iodine-131-sodium iodine (^131^I-NaI); and whole-body oncologic PET scan with ^18^F-fluorodeoxy­glucose (^18^F-FDG). After due appraisal and contemplating the inclusion of 20 candidate patients per diagnosis, we determined that 180 patients should be enrolled in this prospective study.

The inclusion criteria were being ≥ 18 years of age and having no difficulty in urinating. Hospitalized patients were excluded, as were those who were using a urinary catheter and those who were undergoing dialysis.

### Patient radiation dose monitoring and whole-body radiopharmaceutical clearance

The radiation doses (in µSv.h^-1^) emitted by radioactive patients at 1.0 m and 2.0 m were monitored with a precalibrated Geiger-Müller detector (MIR-7028; MRA-Equipamentos e Serviços para Radioproteção, Ribeirão Preto, SP, Brazil). Dose rate measurements were carried out by placing the detector in front of the patient (standing erect), at 1.0 meter above ground level. The detector had been calibrated with the standard radioactive sources cobalt-60 and cesium-137, with a margin of error of ± 10%. The first dose rate measurements were taken just after radiopharmaceutical administration and before bladder voiding, so as to guarantee that they corresponded to 100% of the activity of the radiopharmaceutical administered, as well as to facilitate the definition of the correlations between the dose rates and the unit of activity incorporated (µSv.h^-1^.MBq^-1^). The residual activity in the syringe was calculated in accordance with ideal correlations between the real amount of activity injected and the dose emitted by the patient.

**Figure 1:**
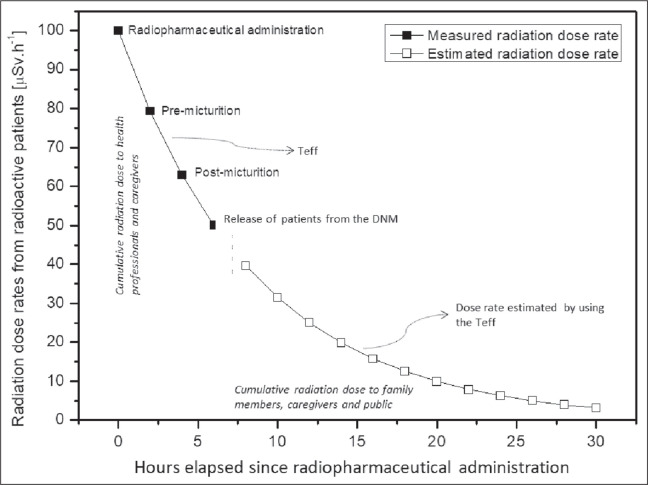
Methodology used for radiation dose evaluation in radioactive patients.

Periodic measurement was the form chosen for monitoring patient radiation dose rates, as well as for estimating evolution of the remaining whole-body radiopharmaceutical activity at various time points after radiopharmaceutical administration: immediately after radiopharmaceutical administration; immediately before micturition and image acquisition; immediately after micturition but before image acquisition; and after image acquisition but prior to patient release from the Department of Nuclear Medicine (DNM). Evaluating the patients at those four time points facilitated the investigation of the gradual reduction of radiation emissions into the surroundings (Figure 1). To minimize the influence of background radiation during dose rate measurement, all measurements were made inside a 2.0 × 2.5 m radiopharmaceutical administration room, with 27 cm-thick concrete walls. Each data point for the dose emitted from the patients over time corresponded to the average of a set of three measurements, after subtraction of the previously determined level of background radiation.

The amount of whole-body radiopharmaceutical activity, cumulatively since administration and sequentially at the four time points, was estimated according to the correlations between the patient dose rate at 2.0 m at that time and the unit of activity injected (µSv.h^-1^.MBq^-1^), determined at the first time point. The distance of 2.0 m was used as the reference standard, as previously described^(8)^. Using the same methodology and the dose rates acquired immediately before and after patient micturition, we calculated the amount of radionuclide activity excreted by patients in the first voiding procedure (as a percentage of the total).

Because the dose rate is proportional to the whole-body radiopharmaceutical activity, these radiometric data facilitate the estimation of the percentage of activity eliminated and retained according to time elapsed since the administration of the radiopharmaceutical. Elimination is at a specific clearance rate, which can be expressed as the whole-body effective half-life (Teff) for each patient and radiopharmaceutical.

To describe the dose rate reduction after radiopharmaceutical administration, we adjusted a simple exponential function:


$$\text{final dose rate}=\text{initial dose rate}\times e^{-\lambda t}$$

where *e* is the Euler’s constant (~ 2.718), λ is 0.693/Teff, and *t* is the time elapsed since radiopharmaceutical administration. Thus, the Teff value for each patient could be calculated when measuring radiation doses at a distance of 2.0 m from their bodies. Exponential function adjustment was performed with Microsoft Excel.

### Cumulative effective dose calculations and radiation safety measures

The estimation of the cumulative effective dose at 1.0 m and 2.0 m from the patient was based on the integration of dose rates measured at those distances over time. After plotting the dose rate as a function of the time elapsed since radiopharmaceutical administration, with Origin PRO 8 SR0 software, version 8.0724 (OriginLab Corporation, Northampton, MA, USA), we calculated the cumulative effective dose by determining the area under the graph: dose rate (in µSv.h^-1^) versus the time elapsed since radiopharmaceutical administration (in h). The total area under the graph is equivalent to the total cumulative effective dose emitted by the patients into their surroundings. That dose was derived from the sum of two components-the first calculated from the start of radiopharmaceutical administration until patient release from the DNM (inside the DNM) and the second calculated from the time of patient release from the DNM until complete cessation of whole-body radiopharmaceutical activity (outside the DNM)-and by using the Teff to project dose rates to surroundings beyond the last measurement time point (Figure 1). The mean values for the dose rates from all patients enrolled in the same diagnostic procedure were used in order to construct the graph for dose rate versus time elapsed since radiopharmaceutical administration.

With the cumulative effective doses obtained, we conducted a comparative study of those doses and the limits established by the International Commission on Radiological Protection (ICRP) and the International Atomic Energy Agency (IAEA) for health professionals, family members, volunteers, and the general public^(9,10)^. On the basis of the magnitude of the estimated radiation doses, radiation protection measures will be indicated for diagnostic procedures, with the aim of reducing the potential for radioactive patients to expose others in their surroundings.

### Statistical analysis and ethics

The results are presented as means and standard deviations, with ranges, as necessary. The study was approved by the Research Ethics Committee of the Hospital das Clínicas da Faculdade de Medicina da Universidade de São Paulo (Reference no. 16497/2017), and all participating patients gave written informed consent.

## RESULTS

A total of 175 individuals (95 women and 80 men), with a mean age of 58 ± 14 years (range, 18-82 years), were included in the study. Table 1 presents the number of patients undergoing each diagnostic procedure, as well as the mean activity of the radiopharmaceutical administered. During the data collection period, the demand on our facility for brain scintigraphies with ^99m^Tc-MIBI decreased. Therefore, only five patients were available for inclusion in that group. Table 2 shows the mean dose emitted by patients, as measured at distances of 1.0 m and 2.0 m and per unit of activity. Table 3 presents the whole-body radiopharmaceutical clearance rate (represented by the Teff) and the whole-body activity remaining after release from the DNM. Table 4 shows the cumulative radiation doses inside and outside the DNM, together with the total cumulative radiation dose emitted by the patients up until complete elimination of the radiopharmaceutical.

**Table 1:** Number of patients enrolled, together with the activity of the radionuclides administered, in the diagnostic nuclear medicine procedures evaluated.

Diagnostic procedure	Patients (n)	Activity (MBq) Mean ± SD
^99m^Tc-MDP bone scintigraphy	25	917.40 ± 19.34
^99m^Tc-DMSA static renal scintigraphy	21	191.98 ± 14.42
^99m^Tc-DTPA dynamic renal scintigraphy	22	409.52 ± 26.10
^99m^Tc-MIBI whole-body scintigraphy	21	862.60 ± 17.74
^99m^Tc-MIBI myocardial perfusion study^*^	20	383.63 ± 52.87
^99m^Tc-MIBI brain scintigraphy	5	1,120.80 ± 33.54
^18^F-NaF PET bone scan	21	201.31 ± 12.35
^18^F-FDG whole-body PET scan	20	261.59 ± 58.45
^131^I-NaI whole-body scintigraphy	20	114.63 ± 2.83

* For the first injection (one-day protocol).

**Table 2:** Radiation dose rates at 1.0 m and 2.0 m from the patient, as well as at 1.0 m per unit of activity, immediately after radiopharmaceutical administration.

Diagnostic procedure	Initial dose rate (µSv.h 1)		Dose rate at 1.0 m, per unit of activity (µSv.h-1.MBq-1) × 10-2 Mean ± SD
	At 1.0 m Mean ± SD	At 2.0 m Mean ± SD	
^99m^Tc-MDP bone scintigraphy	17.50 ± 2.11	5.83 ± 0.70	1.91 ± 0.23
^99m^Tc-DMSA static renal scintigraphy	13.63 ± 0.99	4.54 ± 0.33	7.10 ± 0.52
^99m^Tc-DTPA dynamic renal scintigraphy	14.37 ± 0.83	4.79 ± 0.28	3.51 ± 0.20
^99m^Tc-MIBI whole-body scintigraphy	15.82 ± 1.57	5.27 ± 0.52	1.83 ± 0.18
^99m^Tc-MIBI myocardial perfusion study^*^	10.15 ± 2.10	3.40 ± 0.75	2.91 ± 0.21
^99m^Tc-MIBI brain scintigraphy	15.76 ± 0.71	5.25 ± 0.24	1.41 ± 0.06
^18^F-NaF PET bone scan	18.09 ± 3.21	6.03 ± 1.07	9.00 ± 1.60
^18^F-FDG whole-body PET scan	24.36 ± 4.83	8.12 ± 1.61	9.26 ± 1.84
^131^I-NaI whole-body scintigraphy	6.83 ± 1.09	2.28 ± 0.36	5.94 ± 0.95

* After the first injection.

**Table 3:** Radiation dose rates immediately after patient release from the DNM, whole-body radiopharmaceutical Teff, and the whole-body activity remaining at release.

Diagnostic procedure	Teff (h) Mean ± SD	Final exposure rate (µSv.h 1)		Whole-body activity at release (MBq) Mean ± SD
		1.0 m Mean ± SD	2.0 m Mean ± SD	
^99m^Tc-MDP bone scintigraphy	2.02 ± 0.72	4.45 ± 1.21	1.48 ± 0.40	232.79 ± 62.92
^99m^Tc-DMSA static renal scintigraphy	4.43 ± 0.55	5.40 ± 0.41	1.80 ± 0.14	76.12 ± 5.92
^99m^Tc-DTPA dynamic renal scintigraphy	1.33 ± 0.19	8.25 ± 1.25	2.75 ± 0.42	234.81 ± 35.86
^99m^Tc-MIBI whole-body scintigraphy	3.95 ± 0.65	14.87 ± 1.17	4.96 ± 0.39	815.06 ± 64.09
^99m^Tc-MIBI myocardial perfusion study	2.61 ± 1.31	25.48 ± 2.85	8.49 ± 0.95	870.97 ± 97.46
^99m^Tc-MIBI brain scintigraphy	3.95 ± 0.65	7.73 ± 0.85	2.58 ± 0.28	550.89 ± 59.79
^18^F-NaF PET bone scan	1.18 ± 0.30	8.35 ± 1.81	2.78 ± 0.60	92.67 ± 20.00
^18^F-FDG whole-body PET scan	1.48 ± 0.32	7.67 ± 2.07	2.56 ± 0.69	82.95 ± 22.36
^131^I-NaI whole-body scintigraphy	11.41 ± 0.02^*^	6.83 ± 1.09	2.28 ± 0.36	115.0 ± 0.6

* Acquired from Willegaignon et al.^(11)^.

**Table 4:** Estimated cumulative radiation doses at 1.0 m from the patient from the procedure until complete elimination of the radiopharmaceutical administered.

Diagnostic procedure	Inside the DNM (µSv) Mean ± SD	Outside the DNM (µSv) Mean ± SD	Total (µSv) Mean ± SD
^99m^Tc-MDP bone scintigraphy	38.41 ± 13.69	13.10 ± 4.67	51.51 ± 18.36
^99m^Tc-DMSA static renal scintigraphy	52.23 ± 6.49	34.27 ± 4.26	86.51 ± 10.74
^99m^Tc-DTPA dynamic renal scintigraphy	12.01 ± 1.72	16.19 ± 2.31	28.20 ± 4.03
^99m^Tc-MIBI whole-body scintigraphy	5.40 ± 0.89	84.53 ± 13.91	89.93 ± 14.80
^99m^Tc-MIBI myocardial perfusion study	55.12 ± 9.56	94.62 ± 47.17	149.74 ± 56.72
^99m^Tc-MIBI brain scintigraphy	45.65 ± 7.51	43.94 ± 7.23	89.59 ± 14.74
^18^F-NaF PET bone scan	17.06 ± 4.33	14.62 ± 3.72	31.68 ± 8.05
^18^F-FDG whole-body PET scan	36.29 ± 7.85	16.68 ± 3.61	52.97 ± 11.45
^131^I-NaI whole-body scintigraphy	-	94.30 ± 0.17	94.30 ± 0.17

**Figure 2:**
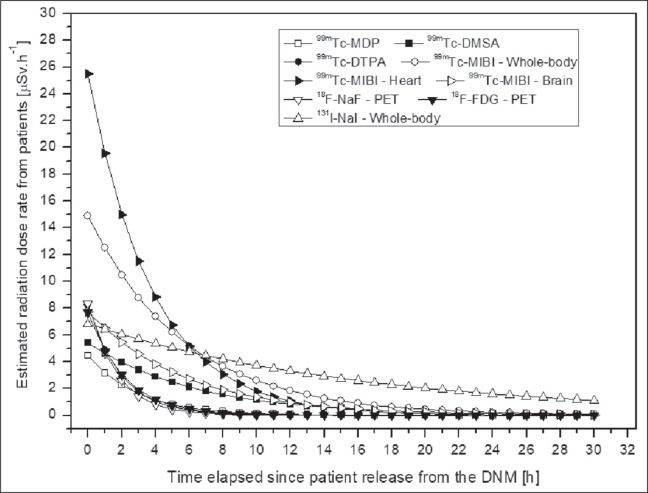
Estimated radiation dose rate from patients after release from DNM.

**Figure 3:**
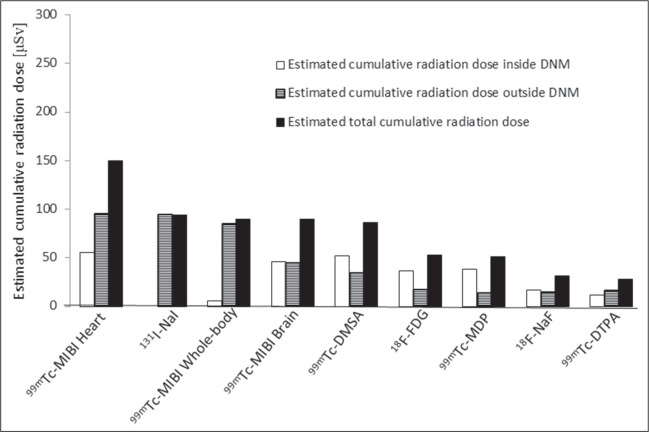
Estimated cumulative radiation doses at 1.0 m from the patient, inside and outside the DNM, by diagnostic procedure.

**Figure 4:**
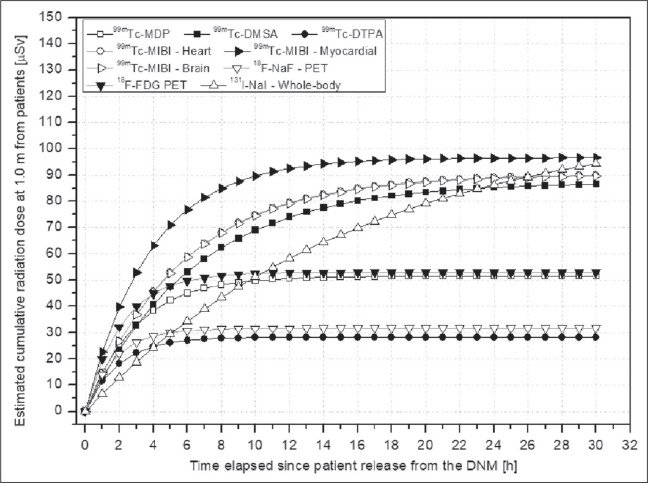
Estimated cumulative radiation doses over time after patient release from the DNM.

The radiation field emitted by patients as a function of time after release from the DNM is shown in Figure 2. The total radiation dose emitted by the patients at 1.0 m, in two periods (inside and outside the DNM), is shown in Figure 3. Figure 4 shows the cumulative radiation dose outside the DNM over time, for all of the radiopharmaceuticals administered.

By using the dose rates obtained immediately before and after patient micturition, we were able to calculate the total radionuclide activity excreted. For ^18^F-NaF, ^99m^Tc-MDP, ^18^F-FDG, ^99m^Tc-MIBI (brain imaging), and ^99m^Tc-MIBI (cardiac imaging) procedures, the respective mean proportions of the total activity were 21% (range, 1.45-35.40%), 17% (range, 8.52-30.79%), 7% (range, 2.61-16.96%), 7% (range, 2.01-9.18%), and 6% (range, 1.64-39.37%). Unfortunately, for the ^99m^Tc-DMSA, ^99m^Tc-DTPA, ^99m^Tc-MIBI (whole-body), and ^131^I-NaI procedures, it was not possible to evaluate excretion, because the clinical examination protocol adopted at our facility did not allow that.

## DISCUSSION

In the present study, we have demonstrated the potential for patients undergoing diagnostic nuclear medicine procedures to expose their surroundings to ionizing radiation after receiving radiopharmaceuticals during diagnostic examinations. The focus was on two periods in the process, during and after the examination. In the first period, mainly health care professionals were exposed, whereas mainly family members, caregivers, and work colleagues were exposed in the second period.

Dose rates obtained for each patient over time were used in order to evaluate the cumulative doses of radiation emitted into their surroundings. Notably, there was individual variation depending on the amount of activity of the radiopharmaceutical administered and on geometric factors. All of the doses obtained immediately after radiopharmaceutical administration and per unit of activity administered were measured at 1.0 m and 2.0 m from the patients.

Our results related to the radiation field emitted by patients as a function of time after release from the DNM are similar to those reported by Stenstad et al.^(7)^ for patients receiving ^99m^Tc-MDP for bone scintigraphy, which were 16 ± 3 µSv.h^-1^ and 6 ± 1 µSv.h^-1^ at 1.0 m and 2.0 m, respectively. Although many studies have investigated the postprocedural radiation doses emitted by individuals receiving radiopharmaceuticals^(3,6,12)^, differences in monitoring procedures, especially in the timing of the measurements, make it difficult to draw comparisons across studies^(13)^.

There is a difference between the radiation dose rates obtained in point- or line-source models and those obtained in real patients. According to Yi et al.^(14)^, those obtained from radioactive patients are approximately 56%, 50%, and 40% lower than are those obtained from ^99m^Tc, ^18^F, and ^131^I point sources, respectively. Willegaignon et al.^(15)^ observed a similar difference for ^131^I sources, also noting a decrease in the dose rate according to the time elapsed since radiopharmaceutical administration. That decrease is worthy of note, given that radiation protection measures related to radioactive patients are typically established according to the potential radiation dose, as presented by point sources, in detriment to those obtained from real patients, as presented in our study.

Based on the adjustment of a simple exponential function to describe the reduction in radiation doses from patients, it was possible to determine the Teff of the radiopharmaceutical for each diagnostic procedure evaluated in the present study. Although the dose rates presented by patients who received positron emitters were higher than were those presented by patients who received other radioisotopes, the radiation field rapidly decreased and, consequently, the potential for exposure was diminished, especially after release of the patient from the DNM. When considering the cumulative radiation dose emitted by patients into their surroundings to a distance of 1.0 m, throughout a diagnostic nuclear medicine examination and after release, we found that the ^99m^Tc-MIBI cardiac imaging procedure (one-day protocol) is capable of producing nearly three times as much radiation exposure as the comparable ^18^F-FDG procedure. That is to be expected, given the shorter physical half-life associated with the latter (1.8 h, compared with 6.0 h for ^99m^Tc-MIBI) as well as the rapid whole-body excretion of ^18^F-FDG.

Of the total radiation dose generated by patients, 50% is emitted into their surroundings while inside the DNM, the remainder being emitted outside the facility. Those proportions vary from procedure to procedure, depending on certain factors, such as the time spent inside the nuclear medicine facility, the amount of radionuclide activity received, and the Teff of the radiopharmaceutical administered. For example, in the whole-body procedure with ^131^I-NaI, nearly all of the radiation dose is emitted outside the facility. However, in diagnostic procedures that involve the administration of positron emitters, most of the patient-emitted radiation dose affects the nearby health professionals, due to the low Teff of the radiopharmaceutical and the longer duration the diagnostic examination.

The likelihood of patients exposing others to radiation after their release from a nuclear medicine facility is basically restricted to the first 24 h after the examination, or even less depending on the radiopharmaceutical administered. In the case of diagnostic examinations employing positron emitters, such as ^18^F-FDG and ^18^F-NaF, which have relatively short physical half-lives, 90% of all patient-emitted radiation is emitted during the first 6 h after the examination, arriving at near zero within the first 24 h. For radiopharmaceuticals including ^99m^Tc radioisotopes, those values are approximately 50% and 97%, respectively, during the first 6 h and 24 h after the examination. Therefore, patients receiving positron emitters for diagnostic examination are less likely to expose others after being released from the nuclear medicine facility than are those receiving radioisotopes such as ^99m^Tc and ^131^I, even if the physical half-life of the radioisotope administered, in doses (major restrictive scenario), is taken into consideration.

In the present study, the highest cumulative radiation doses emitted by the patients into their surroundings after release from the DNM were after ^99m^Tc-MIBI cardiac imaging: approximately 149 µSv at 1.0 m. Even if we consider shorter distances (e.g., 0.5 m), by using the inverse square law, the estimated dose would be 596 µSv, which is approximately half the limit established for the general public (1.0 mSv), and approximately one tenth of that established for family members and caregivers (5.0 mSv per procedure). This is vital when considering protective measures, given that the dose is well below the safe levels stipulated by the ICRP^(9)^ and IAEA^(10)^, even in exceptional cases (e.g., that of someone remaining close to the patient 24 h a day until complete, whole-body elimination of all radiopharmaceuticals). Therefore, appropriate restrictive safety measures are required in order to be in compliance with international radiation protection recommendations. When routinely applied to radioactive patients inside treatment facilities, such measures would be sufficient to guarantee radiation safety.

In the present study, reference values for radionuclide activity excreted by patients in the first micturition after radiopharmaceutical administration were evaluated for ^18^F-NaF, ^99m^Tc-MDP, ^18^F-FDG, ^99m^Tc-MIBI brain imaging, and ^99m^Tc-MIBI cardiac imaging procedures. However, the observed values varied greatly across patients. Such differences could be attributed to the amount of water ingested, the time elapsed between radiopharmaceutical administration and micturition, the clinical stage of the disease, and individual intrinsic radiopharmaceutical biokinetics.

Internal exposure to radioactive substances eliminated by patients is another noteworthy point. The most serious scenario is that associated with lactating patients. In a study analyzing 16 different radiopharmaceuticals in the breast milk of women who had undergone nuclear medicine examinations, with the aim of evaluating the potential for transmitting radiation doses to infants, it was shown that when injected with ^131^I-NaI for diagnostic examinations, the patients needed to interrupt lactation for 12 h after release from the nuclear medicine facility, whereas that was not necessary when the radiopharmaceutical administered was ^99m^Tc-MDP, ^99m^Tc-DMSA, ^99m^Tc-MIBI, or ^18^F-FDG^(16)^. In another study, analyzing the internal radiation dose as a consequence of outpatient thyroid cancer therapy^(17)^, it was shown that when ≤ 7,400 MBq of ^131^I-NaI were administered, not all of the contaminated areas inside the home of the patient represented a significant radiation hazard, given that the maximum potential for an internal radiation dose is approximately 0.23 mSv, even under the worst conditions (e.g., ingestion of contaminated objects). If that is so, it is obvious that diagnostic procedures involving less hazardous substances carry less risk. Hence, radiation protection measures for avoiding internal contamination in diagnostic nuclear medicine procedures are typically not required for adult patients and only slightly so in the case of lactating patients receiving ^131^I-NaI. In addition, the development of novel technologies for radiation detection and new materials for clinical use will facilitate the indication of lower dosages of radiopharmaceuticals, while producing clinical images of the same or even better quality, thereby reducing overall radiation exposure.

The present study served two purposes: to demonstrate the real potential of patients undergoing diagnostic nuclear medicine procedures for exposing others to radiation doses after receiving radiopharmaceuticals; and to present dosimetric data to facilitate the establishment of appropriate radiation safety measures during medical procedures. However, we have shown that, with the exception of cases in which lactating patients receive ^131^I-NaI, such measures are rarely required.

## CONCLUSIONS

Radiation doses emitted by radioactive patients into their surroundings depend on the radiopharmaceutical administered, the amount of activity injected, and the biokinetics of excretion of that radiopharmaceutical from the body. Cumulative radiation doses emitted by patients undergoing diagnostic nuclear medicine procedures are considerably lower than the limits recommended by ICRP and IAEA, which leads us to conclude that precautionary measures to avoid radiation exposure are not required during such procedures, especially after the patient has been released from the nuclear medicine facility.
